# Maintenance Negative Pressure Ventilation Improves Survival in COPD Patients with Exercise Desaturation

**DOI:** 10.3390/jcm8040562

**Published:** 2019-04-25

**Authors:** Hung-Yu Huang, Chun-Yu Lo, Lan-Yan Yang, Fu-Tsai Chung, Te-Fang Sheng, Horng-Chyuan Lin, Chang-Wei Lin, Yu-Chen Huang, Chee-Jen Chang, Kian Fan Chung, Chun-Hua Wang

**Affiliations:** 1 Division of Pulmonary and Critical Care, Department of Internal Medicine, Saint Paul’s Hospital, Taoyuan 330, Taiwan; compaction71@gmail.com (H.Y.H.); naturewei@cgmh.org.tw (C.-W.L.); 2 Department of Thoracic Medicine, Chang Gung Memorial Hospital, Taipei 105, Taiwan; mixova@yahoo.com (C.Y.L.); vikingchung@yahoo.com.tw (F.-T.C.); rubysheng050@gmail.com (T.-F.S.); lin53424@ms13.hinet.net (H.-C.L.); yuchenhahaha@gmail.com (Y.C.H.); 3 College of Medicine, Chang Gung University, Taoyuan 333, Taiwan; 4 Biostatistics Unit, Clinical Trial Center, Chang Gung Memorial Hospital, Taoyuan 333, Taiwan; lyyang0111@gmail.com; 5 Research Services Center for Health Information, Chang Gung University, Taoyuan 333, Taiwan; cjchang@mail.cgu.edu.tw; 6 Experimental Studies, National Heart & Lung Institute, Imperial College London & Biomedical Research Unit, Royal Brompton Hospital, London SW3 6LY, UK; f.chung@imperial.ac.uk

**Keywords:** chronic obstructive pulmonary disease, maintenance negative pressure ventilation, desaturation, 6-min walk test, mortality

## Abstract

Negative pressure ventilation (NPV), when used as an adjuvant to pulmonary rehabilitation, improves lung function, increases exercise capacity, and reduces exacerbations. The aim of this study was to determine whether maintenance NPV improves long-term clinical outcomes and reduces mortality in patients with chronic obstructive pulmonary disease (COPD). Between 2003 and 2009, 341 patients were treated for COPD either with or without hospital-based NPV. We measured forced expiratory volume in one second (FEV_1_), 6-min walking distance (6MWD), and oxygen saturation by pulse oximetry (SpO_2_) during a 6-min walk test (6MWT) every 3–6 months. Desaturation (D) during the 6MWT was defined as a reduction in SpO_2_ of ≥10% from baseline. The NPV group had a better survival outcome than the Non-NPV group. The 8-year survival probabilities for the NPV and Non-NPV groups were 60% and 20%, respectively (*p* < 0.01). Baseline desaturation was a significant risk factor for death, and the risk of death increased with desaturation severity (SpO_2_ 80~89: hazard ratios (HR) 2.7, 95% confidence interval (CI) 1.4–5.3; SpO_2_ < 80: HR 3.1, 95% CI 1.3–7.4). The NPV group had a slower decline in lung function and 6MWD. The NPV + D and Non-NPV+D had a threefold and fourfold increase in the risks of all-cause mortality compared with the NPV-ND, respectively. Maintenance non-invasive NPV reduced long-term mortality in COPD patients. The desaturating COPD patients had an increased mortality risk compared with non-desaturating COPD patients.

## 1. Introduction 

Chronic obstructive pulmonary disease (COPD) is characterized by cellular inflammation accompanied by extensive airway remodeling and parenchymal destruction, which lead to breathlessness and chronic airflow obstruction [1]. The six-minute walk test (6MWT) has been used to assess the functional performance of patients with COPD [2], and oxygen desaturation during the 6MWT has been reported to predict mortality [3,4,5] and increase the risk of adverse outcomes [6].

The mechanism underlying desaturation during the 6MWT is multifactorial and involves dynamic hyperinflation and impaired gas exchange that worsen ventilation-perfusion mismatch [7,8]. Pharmacological and non-pharmacological interventions to decrease lung hyperinflation tend to improve the total lung capacity, which is associated with the duration of exercise among COPD patients [9]. Negative pressure ventilation (NPV) has been shown to be effective in improving ventilation inhomogeneity and gas exchange and in unloading inspiratory muscles, thereby decreasing the work of breathing [10,11]. We previously established a hospital-based maintenance pulmonary rehabilitation program that included NPV for patients with COPD [12,13], and we showed that NPV improved lung function and exercise capacity and reduced acute exacerbation rates [13].

The presence of emphysema with high residual volume has been reported to be an independent risk factor for mortality in patients with COPD [14]. Therefore, any intervention such as pulmonary rehabilitation that does not improve dynamic hyperinflation or exertional desaturation will have no effect on mortality [15,16,17,18]. Accordingly, the aim of this study was to investigate whether long-term regular maintenance NPV adjuvant to pulmonary rehabilitation can improve clinical outcomes and reduce mortality in COPD patients with or without desaturation during the 6MWT. 

## 2. Materials and Methods

### 2.1. Study Population

This was a retrospectively longitudinal observational study. Participants were recruited from the outpatient clinic of Chang Gung Memorial Hospital between 2003 and 2009. There were 421 COPD patients who were confirmed to have airflow obstruction by spirometry with a post-bronchodilator ratio of forced expiratory volume in one second (FEV_1_) to forced vital capacity (FVC) that was <0.7 [19]. Exclusion criteria were (1) a previous diagnosis of severe heart failure, malignancy, end-stage renal disease, severe liver cirrhosis, musculoskeletal deconditioning with an exercise performance limitation and (2) the inability to perform baseline 6MWT or routine 6MWT follow-up. Desaturation during the 6MWT was defined as a reduction in oxygen saturation (ΔSpO_2_) of more than 10% of baseline SpO_2_, and the lowest SpO_2_ recorded during 6MWT was used for analysis. The study was approved by the Chang Gung Memorial Hospital Ethics Committee (IRB number: 102-4532B).

### 2.2. Maintenance Negative Pressure Ventilation (NPV) Program 

**Figure 1:**
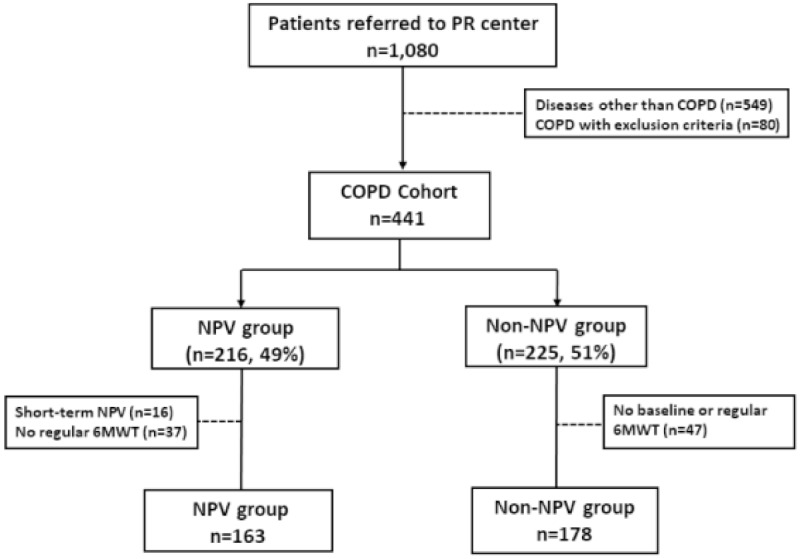
Study flowchart. Abbreviations: COPD: chronic obstructive pulmonary disease; PR: pulmonary rehabilitation; 6MWT: 6-min walk test.

All patients were referred to the Pulmonary Rehabilitation Center of the Thoracic Department of Chang Gung Memorial Hospital. A pulmonary physician explained the maintenance NPV program to the patients and their family. We established a hospital-based maintenance NPV program that included NPV support, breathing training, and an educational program (relaxation techniques and a home pacing walking exercise) in daily clinical practice. Breathing training consisted of breathing techniques (pursued-lipped, controlled, and diaphragmatic breathing). The patients received NPV with breathing training via a cuirass ventilator (cuirass diameter 21 cm or 34 cm, Dima Italia Srl., Bologna, Italy) for 60 min. The patients were not supplemented with oxygen during the application of NPV, except the patients whose baseline saturation was less than 90%. All of the patients were encouraged to perform an unsupervised home exercise program consisting of 20–30 min of a pacing walk with a fixed speed either continuously or intermittently (in sessions lasting 10 or more minutes). The patients were advised to reach 40–60% of the target heart rate or a score of 4–6 on the modified Borg scale and to aim for achieving 3–5 exercise sessions per week. Supplemental oxygen was given to maintain oxygen saturation if desaturation (SpO_2_ < 90%) was observed. The pulmonary rehabilitation program consisted of generic exercise, education, and physical activities. A physiotherapist supervised or trained the patients on the program (relaxation techniques and at-home pacing walk) for 20–30 min at each visit. Initially, we chose an appropriately sized shell for the patient. During the pressure adjustment process, an oximeter was used to record the heart rate and blood oxygen saturation. The level of end-tidal CO_2_ (EtCO_2_) was measured using a nasal EtCO_2_ monitor. The initial negative pressure setting started from −20 cmH_2_O, and if the patient could tolerate or was comfortable with this setting, then the pressure was increased by −5 cmH_2_O every five minutes until it reached −35 cmH_2_O. If the patient could not tolerate or was uncomfortable with the pressure, it was set to the previous level. None of the patients had any adverse effects during the titration period. The NPV ventilator setting (rate, pressure) was adjusted to maintain EtCO_2_ at less than 20% of baseline during the NPV program to ensure the effectiveness of lung expansion therapy. The ventilator was set to the control model with a frequency of 12~15 cycles/min, 30% of the ratio of inspiratory time to total breathing cycle time (Ti/Ttot), and delivery of negative pressures ranging from −20 to −35 cm H_2_O. During the NPV titration process, EtCO_2_ and heart rate significantly decreased, and SpO_2_ was maintained at over 90% (Figure S1). The transition dyspnea index (TDI) was rated in a range from −3 (major deterioration) to +3 (major improvement) [20] and recorded after the patients had undergone NPV for 60 min at the final adjusted pressure. A pattern of response confirmed a 1-unit change in the TDI score as being clinically important [20]. At least a 1-unit improvement in TDI at six days was improved after patients underwent NPV for 60 min (Figure S2). Therefore, the patients in the NPV group underwent the hospital-based NPV once per week in the maintenance program at least three times per month. We had a record of each session of NPV therapy in the Pulmonary Rehabilitation Center, and patients were excluded if they had poor compliance with NPV therapy. The patients who did not wish to enter the hospital-based NPV program were enrolled in the Non-NPV group. These patients gave authorization to be studied but did not use NPV, and they were continuously followed up at outpatient clinics of our hospital for medical treatment every 3 months. The 6MWT was performed every 3–6 months. When they returned to the hospital, either at each outpatient clinic or for performing the 6MWT, they were referred to the Pulmonary Rehabilitation Center and made aware of and be trained for the program of breathing training, relaxation techniques, and the at-home pacing walk exercise for 20–30 min under a physiotherapist’s supervision. The sole difference in treatment between the NPV and the control groups was the application of NPV. Seventeen patients from the non-NPV group were unwilling to perform the 6MWT, and 30 of the non-NPV group and 37 of the NPV group were lost to follow-up after several visits. In addition, 16 patients could not continue the NPV program because of a lack of family support or change in residence. They were excluded for having incomplete medical records. Thus, the remaining 341 consecutive participants completed the 6MWT and had high compliance with treatments as confirmed by medical records. These 341 patients were categorized into two groups: (1) the NPV group (*n* =163) and (2) the Non- NPV group (*n* =178) (Figure 1). 

### 2.3. Clinical Assessment

The main aim of the study was to evaluate the efficacy of long-term regular maintenance NPV adjuvant to COPD therapy for patients who were desaturating or non-desaturating during the 6MWT. Efficacy of the tested treatment was evaluated the effect of treatment on mortality as the main outcome. All subjects performed the 6MWT every 3–6 months. Each participant was instructed to walk back and forth in a 35 m corridor and to stop walking after 6 min, in accordance with the American Thoracic Society recommendations [2]. The six-minute walking distance (6MWD) was then determined. Pulmonary function tests, including FVC, FEV_1_, and the FEV_1_/FVC ratio, were performed before and after walking using a spirometer (ST-250, Fukuda Sangyo Co. Ltd., Nagareyama, Japan). Oxygen saturation was measured during the procedure by finger pulse oximetry (Criticare Systems Inc., Waukesha, WI, USA). 

The survival status and causes of death were ascertained by using the National Register of Deaths provided by the Health and Welfare Data Science Center, Ministry of Health and Welfare in Taiwan. Causes of death included all-cause mortality (International Statistical Classification of Diseases (ICD)-9 codes: 001–998) and disease-specific death categories, including pulmonary diseases (ICD-9 codes: 460–519; ICD-10 codes: J00-J98), lung cancer (ICD-9 codes: 140–239; ICD-10 codes: C00-D49), cardiovascular diseases (ICD-9 codes: 390–459; ICD-10 codes: I00-I87), and other diseases. Previous studies have validated the accuracy of the ICD coding in the Taiwan National Deaths Registry [21,22]. 

Secondary efficacy points included the frequency of acute exacerbations and hospitalizations and data obtained from the 6MWT. The data recorded for each patient included the yearly decline in FEV_1_, 6MWD, and distance-saturation product. The distance-desaturation product was the 6MWD multiplied by the lowest SpO_2_ during the 6MWT. Exacerbations were defined as a period of worse respiratory symptoms that needed treatment with corticosteroids or antibiotics or both [23]. Medical records were inspected following the completion of the NPV program to determine the timing and incidence of exacerbations that led to an emergency room (ER) visit and/or hospitalization. An ER visit was defined as an ER stay of >18 h that required treatment for increased respiratory symptoms [13,23]. Hospitalization was defined as ward admission for the treatment of COPD exacerbation [19].

### 2.4. Sample Size Calculation

Sample sizes of the NPV (*n* = 163) and the Non-NPV (*n* = 178) groups achieved an 82% power to detect a difference between group mortality proportions of 14.6% on the basis of the two-proportion equality test with unpooled variance at 5% significance level. The mortality rate was assumed to be 41.6% in the Non-NPV group and 27% in the NPV group.

### 2.5. Statistical Analysis

Descriptive statistics were used to summarize patient characteristics. Comparisons between groups were performed using analysis of variance for continuous variables and a chi-square test for categorical variables. The Kruskal–Wallis test was used when the assumptions of one-way analysis of variance were not met. Overall survival was defined as the time from the date of enrollment to the date of death or censored at the last follow-up visit for patients still living. Cumulative survival curves were plotted using the Kaplan–Meier method and compared with the log-rank test. Multivariate Cox regression models were used to identify the predictors of overall survival. The results were expressed as hazard ratios (HRs) with their 95% confidence intervals (CIs). For pulmonary functions, 6MWD, and distance-saturation products (DSP, M%) that were not performed at regular time-points, a mixed-model repeated-measure analysis was used for subjects with repeated scheduled measurements to examine changes in pulmonary function and 6MWD and to compare the differences between groups. Statistical analyses were performed using SAS 9.2 (SAS Institute, Cary, NC, USA). A *p*-value < 0.05 was considered to be statistically significant.

## 3. Results

### 3.1. NPV Program Improved Survival in the COPD Patients

**Figure 2:**
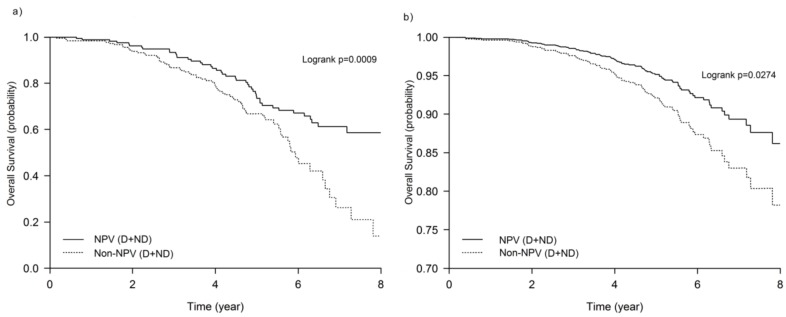
Kaplan–Meier survival curves for the effect of maintenance negative pressure ventilation (NPV) on 8-year all-cause mortality of the cohort: (**a**) unadjusted, (**b**) after adjustment for sex, age, body mass index, smoking, Charlson comorbidity score, forced expiratory volume in the first second at baseline, exacerbations rate, and 6-min walk distance. Significance was determined by using the log-rank test. Abbreviations: D: desaturation, ND: non- desaturation.

The characteristics of the patients are summarized in Table 1. The median follow-up time was 5.8 years in the NPV group and 4.7 years in the Non-NPV group. There were 44 (27.0%) deaths among the NPV group and 74 (41.6%) deaths among the Non-NPV group (*p* = 0.004). The maintenance NPV program improved the overall survival probability of COPD patients (Figure 2). The 8-year survival probabilities for the NPV and the Non-NPV groups were 60% and 20%, respectively (*p* < 0.01). The patients who did not receive maintenance NPV had a 2.5-fold increased risk of mortality at the 8-year follow-up point compared with the NPV + ND group (*p* = 0.03). There was no significant difference in adjusted medications during the follow-up period between the NPV and Non-NPV groups. There were also no significant differences in the appearance of comorbidities over the follow-up period between the two groups.

### 3.2. Desaturation Associated with Lower Lung Function and Higher Mortality

Overall, 83 patients (50.9%) in the NPV group and 93 patients (52.2%) in the Non-NPV group showed desaturation during the 6MWT. The desaturators had a lower FEV_1_, lower BMI, and higher GOLD stages than the non-desaturators regardless of whether or not they were included in the NVP program (Table 2). There were no significant differences in age, gender, 6MWD, therapy changes, or appearance of comorbidities (such as ischemic heart disease, cerebrovascular disease, diabetes, liver disease, and chronic kidney disease) among these four groups. The desaturators had higher all-cause mortality rates (NPV+D: 37.3%; Non-NPV+D: 48.3%) compared with the non-desaturators (NPV + ND: 16.2%; Non-NPV + ND: 34.1%, *p* = 0.008). A pulmonary cause was the major cause of death, and other causes of mortality were similar among the four groups. When adjusted by sex, age, smoking status, FEV_1_, hospitalization, and Charlson index in the multivariate analysis, 6MWD and desaturation during the 6MWT were predictive of mortality (Table 3). Desaturation at baseline 6MWT was a significant risk factor for death, and the risk of death increased with desaturation severity [SpO_2_ 80~89: hazard ratio (HR) 2.7, 95% confidence interval (CI) 1.4–5.3; SpO_2_ < 80: HR 3.1, 95% CI 1.3–7.4]. 

### 3.3. Higher Mortality Rate of the Desaturators Improved by the Maintenance NPV Program

**Figure 3:**
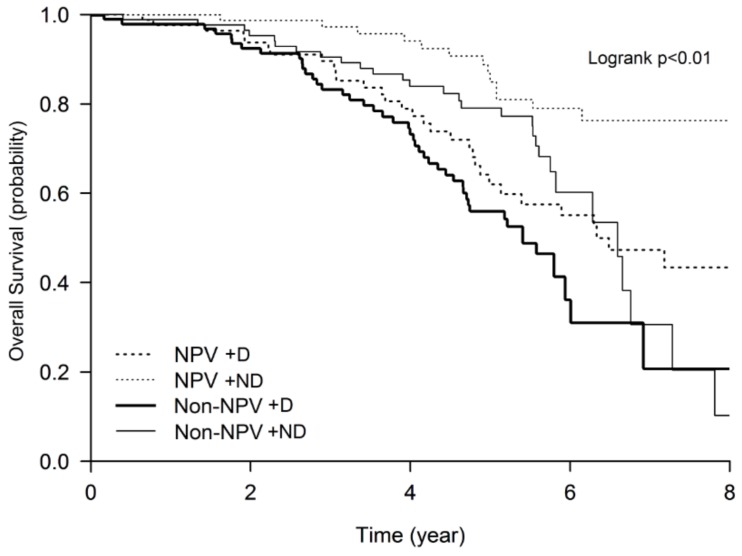
Kaplan–Meier survival curves for the effect of maintenance negative pressure ventilation (NPV) and desaturation on the 8-year all-cause mortality of the cohort. Abbreviations: D: desaturation, ND: non-desaturation. The *p*-value is indicated.

Kaplan–Meier plots revealed significant differences in survival trajectories among all four groups (log-rank test, *p* < 0.05), and the survival rates progressively decreased from the NPV + ND group to the Non-NPV + D group (Figure 3). Relative to the NPV + ND group (reference group), the risk of mortality at the 8^th^ year was significantly increased in all of the other groups (Figure 3). The NPV + D (HR 3, 95% CI 2–7) and Non-NPV + D (HR 4, 95% CI 2–7) groups had a threefold and fourfold increased risk of all-cause mortality at the 8th year of follow-up compared with the NPV + ND group, respectively (Table 3). The risks of mortality during the 8 years in the four groups, adjusted by confounders at 3, 5, and 8 years, are shown in supplementary Table S1.

### 3.4. Exacerbations and Hospitalizations

**Figure 4:**
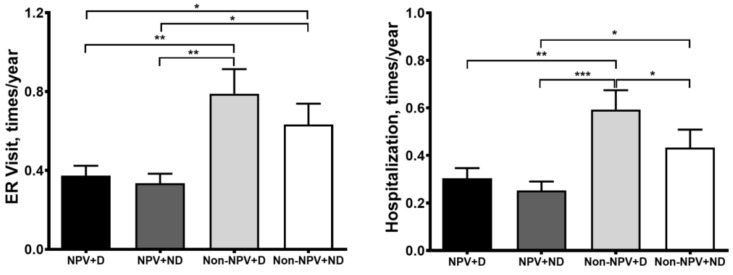
The frequency of emergency room (ER) visits and hospitalization rates is indicated as times per year. The *p* value is indicated. * *p* < 0.05, ** *p* < 0.01, *** *p* < 0.001.

The number of ER visits per person-year in the 4 groups was 0.4 (NPV + D), 0.3 (NPV + ND), 0.8 (Non-NPV + D), and 0.6 (Non-NPV + ND) (*p* < 0.01 Kruskal–Wallis test), respectively. The mean number of hospitalizations in the Non-NPV + D group (0.6 per person-year) was higher than that of the NPV + D group (0.3 per person-year, *p* = 0.001). The number of ER visits and hospitalizations per person-year was not different between the NPV + D and NPV + ND groups (Figure 4). 

### 3.5. Longitudinal Changes in Lung Function, Walking Distance, and Hypoxia Index

**Figure 5:**
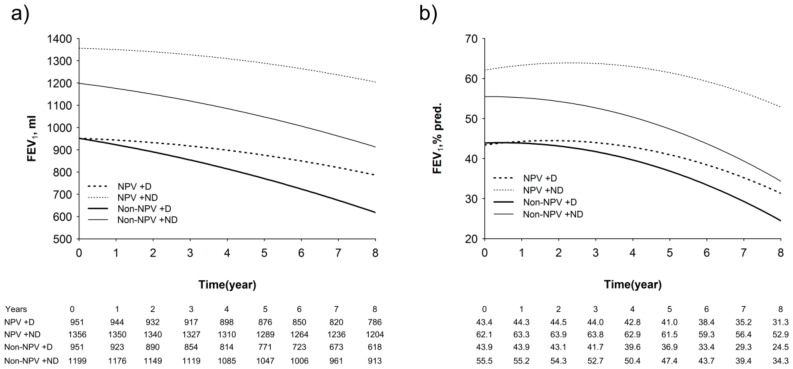
Modeled forced expiratory volume in one second (FEV_1_) over time in volume of FEV_1_ (mL) (**a**) and in predicted value of FEV_1_ (pred.%) (**b**). Abbreviations: D: desaturation, ND: non-desaturation.

The Non-NPV groups had a significantly greater decline in both FEV_1_ and FEV_1_% predicted values than the NPV groups (Figure 5a,b). The estimated annual decline in FEV_1_ was 19 mL in the NPV + ND group, 20 mL in the NPV + D group, 35 mL in the Non-NPV + ND group, and 42 mL in the Non-NPV + D group. The detailed analysis of the mixed-model repeated-measure models is shown in Supplementary Table S2.

**Figure 6:**
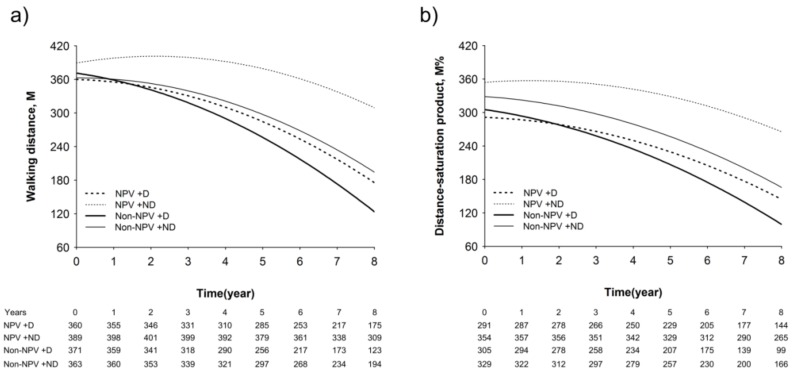
Modeled 6-min walk distance (meter, M) (**a**) and distance-saturation product (M %) (**b**) over time.

In the 6MWD model, the groups had different trajectories of decline (Figure 6). The NPV + ND group showed an improvement in walking distance at 1 year (398.0 m), 2 years (401.3 m), and 3 years (399.3 m), and it then began to decrease from the fifth year (379.2 m) compared with baseline (389.3 m) (Figure 6a). However, in the Non-NPV group, both desaturators and non-desaturators displayed a rapid decline in the 6MWD during the 8 years (Non-NPV + D, from 371.1 to 123.4 m; Non-NPV + ND, from 326.6 to 194.2 m) (Figure 6a). The slope of the 6MWD over time in the NPV + ND group was significantly lower compared with that of the Non-NPV group (*p* < 0.0001) (Figure 6a). The interaction effects of time and group (*p* = 0.003), time (*p* = 0.014), and the quadratic time (*p* < 0.001) were significantly different between the four groups. The DSP, which included surveillance of both the walking distance and the lowest oxygen saturation of the patients during the 6MWT, was a predictive factor of a worsening condition and survival. The slope of the DSP over time in the NPV + ND group was significantly lower compared with the Non-NPV group (*p* < 0.0001), Non-NPV + ND (*p* = 0.005), and NPV + D (*p* = 0.0009) groups (Figure 6b). The effects of interaction of time and group (*p* < 0.0001), time (*p* = 0.001), and the quadratic time (*p* < 0.0001) were significant among the four groups (Supplementary Tables S3 and S4).

## 4. Discussion

We show for the first time that a hospital-based maintenance NPV program reduced mortality and acute exacerbations and hospitalizations; we also show the decline in lung function and walking distance in patients with COPD. Patients who desaturated during the 6MWT at the study baseline had a four-fold increased risk of death, together with a more rapid rate of decline in FEV_1_ and walking distance, factors that are associated with a poor mortality prognosis [4,6]. However, the maintenance NPV program reduced long-term mortality in COPD patients irrespective of the presence of desaturation during the 6MWT. 

Noninvasive ventilation with bilevel positive airway pressure (BiPAP) has also been reported to reduce lung hyperinflation and inspiratory loads [24] and to improve FEV_1_, the 6MWT, and transition dyspnea index in hypercapnic COPD patients [25]. Daily BiPAP use for one year also reduced mortality in hypercapnic COPD patients [26]. A retrospective analysis revealed that the long-term use of noninvasive positive pressure ventilation in stable COPD patients resulted in decreased hyperinflation by reducing residual volume/total lung capacity (RV/TLC) and improving inspiratory capacity [27]. During titration of NPV pressure in our study, the level of end-tidal CO_2_ (clinical uses determining hypo- or hyperventilation) was decreased according to the increased level of negative pressure (Figure S1). Moreover, the improvement in dyspnea after NPV persevered for 6 days. Therefore, the mechanism of the effect of NPV in our study is still unclear and may in part have resulted from reduced dead space or decreased respiratory muscle load.

Currently, there is little evidence that pulmonary rehabilitation can improve survival in stable COPD patients. The survival rate after rehabilitation has been reported to be 73% or 80% after four or three years, respectively, [17,28] in studies that included patients other than those with COPD. At six years, another study reported a survival rate of 67% for patients undergoing pulmonary rehabilitation compared with 56% for the control group [29]. By comparison, the five-year survival of our study was 80% for the NPV group and 65% for the non-NPV group. The difference may be because the previous studies did not include treatment for improving dynamic hyperinflation and ventilation/perfusion abnormality as included in BiPAP or NPV ventilation. In our current study, even for the non-desaturating COPD patients, NPV adjuvant to pulmonary rehabilitation was a protective factor for mortality. This finding may have important implications for clinical practice: COPD patients with or without desaturating should be advised to receive NPV adjuvant to programs designed to improve survival. 

Desaturation during the 6MWT is an important predictor of faster lung function decline, a high risk of exacerbations, and higher mortality in patients with COPD [6]. We found that desaturators during the 6MWT had a higher mortality rate than non-desaturators in both the NPV and non-NPV groups, and desaturation was an important factor that affected mortality from the fifth year after enrollment. Previous studies measured desaturation by using pre- and post-test SpO_2_, but this factor alone may not appropriately reflect the severity of desaturation [4,6]. Most importantly, we found that the hazard ratio of mortality for a nadir desaturation of less than 80% during the 6MWT was higher than that for a nadir saturation of 80–90%, indicating that the mortality rate was directly proportional to the degree of desaturation. The major contributor to exertional hypoxemia in COPD patients is ventilation/perfusion mismatch resulting from dynamic airflow limitation and destruction of the pulmonary capillary bed [30]. Although the underlying mechanisms for the exertional desaturation associated with mortality in patients with COPD are not fully established to date, the accompanying hypoxia and hypoxemia may promote bacterial infections [31], enhance the activation of hypoxia-inducible factors (HIFs) and nuclear factor (NF)-κB [32], and propagate systemic inflammation or increase the number of recurrent exacerbations [32,33]. Thus, any modality improving exertion-induced hypoxemia may reduce mortality among COPD patients.

The 6MWD represents the exercise capacity of COPD patients. Exertional desaturation is not the only factor that determines 6MWD, and other factors, including muscle power, cardiopulmonary function, balance, and dyspnea, may also influence the result of the 6MWD. Although the lowest and the highest 6MWD values were low in the desaturators (NPV + D: 72~572 m; Non-NPV + D: 120~546 m) compared with the non-desaturators (NPV + ND: 114–612 m; Non-NPV + ND: 120~585 m), the distribution of 6MWD was wide and variable in each group. There was no significant difference in the mean values at baseline because of the wide range of 6MWD values in each group. However, after enrollment into the study, the COPD patients with desaturation had a significantly greater yearly decline in 6MWD. NPV therapy adjuvant to a pulmonary rehabilitation program could slow down the yearly decline in 6MWD, irrespective of exercise desaturation.

Pulmonary rehabilitation using intensive exercise training relieved symptoms and improved exercise capacity during the initial intensive program for patients with COPD [34]; however, worse survival was observed in COPD patients with poor 6MWD results and a lack of improvement [35]. We also found that the improvement in 6MWD was related to mortality in our cohort population. Although the impact of rehabilitation-induced changes in 6MWD on the survival of patients with COPD has not been fully elucidated, one possible explanation is that worse desaturation during walking or exercise may lead to systemic inflammation and worsen emphysematous structural changes, thus augmenting muscle dysfunction and decreasing daily activity. Therefore, the use of bronchodilators, pacing walk with breathing control training, and lung expansion therapy may lead to clinical improvements in exertional desaturation or walking distance and hence decrease the frequency of exacerbations [16,36,37]. This may be the reason that the results of our program revealed a decreased yearly decline in 6MWD associated with improved mortality in the desaturators of COPD patients.

The distance-saturation product (DSP, M%), which indicates both the walking distance and lowest oxygen saturation of patients during the 6MWT, has been proposed to be a predictive factor of a worsening condition and survival for patients with bronchiectasis [38] and idiopathic pulmonary fibrosis [39]. During the 6MWT, the distance walked is highly dependent on the effort of the patients, and limited effort may result in a reduced distance and/or increased time walked, thus avoiding the subsequent development of hypoxia. In addition, greater hypoxia during a walking test may lead to dyspnea and less distance walked. A previous study indicated that DSP may prevent the impact of effort and decrease the interference of the variability of desaturation during the 6MWT and that it may be a more reliable predictive factor of survival in lung disease patients [39]. Our results showed that both walking distance and the lowest oxygen saturation during the 6MWT were independent predictive factors of outcome in our COPD patients. Combining both parameters into a composite index, DSP, provided accurate prognostic information that was similar to that of 6MWD. This index, which is simple to use in clinical practice, provides an estimation of mortality risk and may identify patients who require more intensive management.

We added NPV as an adjunct to pulmonary rehabilitation because it has the advantages of unloading inspiratory muscles, improving breathing patterns, and assisting sputum clearance in the acute setting of COPD [10,11]. We have demonstrated the benefits of NPV in patients with COPD, bronchiectasis, and restrictive lung diseases [12,13]. Thus, our program of maintenance NPV for COPD patients may have the clinical effects of decreasing dead space and inhomogeneous lung ventilation while increasing pulmonary perfusion and clearance of mucus plugging that is associated with lung atelectasis, and these factors could contribute to improved ventilation/perfusion mismatching and shunting and reduced hypoxemia at rest or during exercise. Therefore, NPV as an adjuvant to pulmonary rehabilitation can lead to slowing lung function decline, maintaining exercise capacity, reducing acute exacerbations, and prolonging survival.

### Limitations

There are several limitations to this study. First, as this study was a retrospective analysis, a randomized, multicentric, control study to evaluate the long-term outcome of maintenance NPV as adjuvant therapy for COPD patients would provide further the evidence to complement the results of this study. However, the study results and its performance within clinical practice reflected real-world experiences with less selection bias. Second, since we did not collect information on the physiological parameters of NPV, we could not confirm the mechanism underlying the beneficial effect on mortality. We will conduct a prospective study, including measurements of biomarkers and parameters of hyperinflation. Third, we did not measure arterial blood gases and, therefore, did not collect data on arterial oxygen or carbon dioxide tension. Instead, we only used the nadir SpO_2_ as a marker of desaturation. Fourth, we performed multivariate analysis in an attempt to control the confounder factors impacting mortality. However, we cannot exclude the possibility that at least part of the benefit of lower mortality in the NPV group was due to the greater severity of the disease in those patients in the non-NPV group. Fifth, since there were no significant differences at baseline between adherence to the breathing exercise, educational program, and other treatments between groups, the difference in mortality between the groups could be in part affected by the follow-up maintenance of the breathing exercise, educational program, and adherence to at-home pacing walk. The difference after the follow-up program could not be determined by medical records, thus leading to some bias of mortality between the groups. Further studies are needed to clarify this point. Finally, compliance is a very important factor to evaluate the true effect of NPV on the mortality of COPD patients. Our study did analyze the outcomes of those patients having poor compliance, those patients with initial poor compliance that improved their compliance to treatment over time, or conversely, those patients with good initial compliance and poor compliance throughout the study. Further studies are needed to investigate this issue.

## 5. Conclusions

This is the first report of the simple protocol effect of maintenance non-invasive NPV that demonstrates an improvement in the mortality of COPD patients who presented desaturation during the 6MWT. With the extensive adjustment for important confounders, this study showed a consistent pattern of increased risk of death, worsening lung function, increased acute exacerbations, and reduced walking distance or distance-saturation product among patients who experienced desaturation during the 6MWT. Maintenance non-invasive NPV could improve these parameters. We conclude that the simple protocol effect of maintenance non-invasive NPV reduces long-term mortality and improves clinical outcomes in COPD patients. A randomized controlled trial is needed to confirm these findings.

## Figures and Tables

**Table 1:** Characteristics and mortality outcome.

	NPV	Non-NPV	*p*-Value
	(*n* = 163)	(*n* = 178)	
Age, years	69 ± 9.7	71 ± 8.1	0.018
Gender, male	145 (89%)	167 (93.8%)	0.108
Smoking exposure			0.556
Current smoker	115	137	
Ex-smoker	32	28	
Non-smoker	16	17	
Smoking (PKY)	28.4 ± 33.2	35.1 ± 28.5	0.050
Body mass index, kg/m^2^	22.7 ± 4	22.5 ± 3.7	0.516
GOLD stage			0.999
I	18 (11%)	19 (10.7%)	
II	53 (32.5%)	58 (32.6%)	
III	62 (38%)	69 (38.8%)	
IV	30 (18.4%)	32 (18%)	
Charlson index	3.0 (1.9)	2.7 (1.9)	0.094
Appearance of comorbidities during follow-up			0.712
Ischemic heart disease	24	33	0.346
Cerebrovascular disease	9	8	0.663
Diabetes	26	21	0.266
Liver disease	4	3	0.617
Chronic kidney disease	1	1	0.950
Walking distance (6MWD), M	369.5 ± 107.2	360 ± 100.5	0.400
FVC, L	1.9 ± 0.7	1.8 ± 0.5	0.500
FVC, % pred.	62.6 ± 22.3	60.4 ± 18.8	0.324
FEV_1_, L	1.1 ± 0.5	1.1 ± 0.4	0.162
FEV_1_, % pred.	52.2 ± 23.3	50.2 ± 21.8	0.425
FEV_1_/FVC, %	62.7 ± 38.7	57.3 ± 11.1	0.077
O_2_ saturation			
Pre-exercise, %	94.7 ± 2.7	95 ± 2.4	0.235
During-exercise, %	85.6 ± 7.8	85.5 ± 7.8	0.928
Medications			
At baseline			
LAMA alone	14	8	0.101
LABA + ICS	34	27	
LAMA + LABA + ICS	108	133	
At end of follow-up			0.210
LAMA alone	13	9	
LABA + ICS	36	30	
LAMA + LABA + ICS	112	137	
Overall mortality events			
All-cause death, *n* (%)	44 (27.0)	74 (41.6)	0.006
Cardiovascular death, *n* (%)	4 (2.5)	7 (3.9)	0.440
Lung cancer death, *n* (%)	3 (1.8)	9 (5.1)	0.107
Other death, *n* (%)	9 (5.5)	9 (5.1)	0.848
Disease-specific death (pulmonary, cardiovascular, and lung cancer), *n* (%)	35 (13.3)	65 (37.3)	0.002

Data are shown as mean ± SD, *n* (%), or *p*-value. *Abbreviations*: NPV: negative pressure ventilation, D: desaturation, ND: non-desaturation, PKY: pack per year, BMI: body mass index, 6MWD: 6-min walk distance, FEV_1_: forced expiratory volume in the first second, FVC: forced vital capacity, LAMA: long-acting muscarinic antagonist, LABA: long-acting beta-adrenergic agonist, ICS: inhaled corticosteroid.

**Table 2:** Clinical characteristics and mortality outcome of subgroups.

	NPV + D	NPV + ND	Non-NPV + D	Non-NPV + ND	*p*-Value
	(*n* = 83)	(*n* = 80)	(*n* = 93)	(*n* = 85)	
Age, years	69 ± 9.1	68.7 ± 10.3	71 ± 9.1	71.4 ± 6.8	0.108 ^a^
Gender, male	76 (91.6%)	69 (86.3%)	87 (93.5%)	80 (94.1%)	0.253
Smoking exposure					0.244
Current smoker	63	52	76	61	
Ex-smoker	15	17	12	16	
Non-smoker	5	11	5	8	
Smoking (PKY)	31.3 ± 36.2	25.3 ± 29.6	37.8 ± 31.5	32 ± 24.6	0.078
Body mass index, kg/m^2^	22.3 ± 4	23.2 ± 3.9	21.8 ± 3.8	23.2 ± 3.3	0.049
GOLD stage					<0.001
I	3 (3.6%)	15 (18.8%)	6 (6.5%)	13 (15.3%)	
II	17 (20.5%)	36 (45%)	21 (22.6%)	37 (43.5%)	
III	37 (44.6%)	25 (31.3%)	42 (45.2%)	27 (31.8%)	
IV	26 (31.3%)	4 (5%)	24 (25.8%)	8 (9.4%)	
Charlson index	3.2 ± 2.1	2.8 ± 1.8	2.6 ± 1.7	2.8 ± 2.1	0.260
Appearance of comorbidities during follow-up					0.586
Ischemic heart disease	14	10	19	20	0.293
Cerebrovascular disease	8	1	6	2	0.051
Diabetes	18	8	10	11	0.107
Liver disease	3	1	1	2	0.627
Chronic kidney disease	1	0	1	0	0.584
Walking distance (6MWD), M	357.6 ± 114	381.8 ± 98.9	363.8 ± 98.4	355.9 ± 103.1	0.366
FVC, L	1.6 ± 0.5	2.1 ± 0.7	1.7 ± 0.5	2 ± 0.6	<0.001 ^a^
FVC, % pred.	54.1 ± 18.3	71.4 ± 22.7	55.9 ± 16.8	65.3 ± 19.7	<0.001 ^a^
FEV_1_, L	0.9 ± 0.4	1.3 ± 0.5	0.9 ± 0.4	1.2 ± 0.5	<0.001 ^a^
FEV_1_, % pred.	43.3 ± 19.3	61.4 ± 23.5	44.1 ± 18.5	57 ± 23.2	<0.001 ^a^
FEV_1_/FVC, %	63.3 ± 13.2	62 ± 11.4	54.7 ± 11.4	60.2 ± 10.1	0.175
O_2_ saturation					
Pre-exercise, %	93.5 ± 3.0	95.9 ± 1.8	94.5 ± 2.8	95.6 ± 1.7	<0.001 ^a^
During-exercise, %	80.6 ± 7.4	90.8 ± 3.8	80.3 ± 7.2	91.2 ± 2.6	<0.001 ^a^
Medications					
At baseline					0.574
LAMA alone	7	7	4	4	
LABA + ICS	18	16	15	12	
LAMA + LABA + ICS	58	50	70	63	
At end of follow-up					0.376
LAMA alone	7	6	5	4	
LABA + ICS	14	22	16	14	
LAMA + LABA + ICS	62	50	72	65	
Overall mortality events					
All-cause death, *n* (%)	31 (37.3)	13 (16.2) *	45 (48.3)	29 (34.1)	0.0001
Pulmonary death, *n* (%)	22 (26.5)	6 (7.5)	35 (37.6)	14 (16.5)	<0.0001
Cardiovascular death, *n* (%)	2 (2.4)	2 (2.5)	2 (2.2)	5 (5.9)	0.4613
Lung cancer death, *n* (%)	1 (1.2)	2 (2.5)	4 (4.3)	5 (5.9)	0.3735
Other death, *n* (%)	6 (7.2)	3 (3.8)	4 (4.3)	5 (5.9)	0.7422
Disease-specific death (pulmonary, cardiovascular and lung cancer), *n* (%)	25 (30.1)	10 (12.5) *	41 (44.1)	24 (28.2)	0.0001

Data are shown as mean ± SD, *n* (%), or *p* value. *Abbreviations*: NPV: negative pressure ventilation, D: desaturation, ND: non-desaturation, PKY: pack per year, BMI: body mass index, 6MWD: 6-min walk distance, FEV_1_: forced expiratory volume in the first second, FVC: forced vital capacity, LAMA: long-acting muscarinic antagonist, LABA: long-acting beta-adrenergic agonist, ICS: inhaled corticosteroid; *p*-value was based on one-way ANOVA or Chi-square test.*p < 0.05 versus Non-NPV + ND. ^a^ Welch’s ANOVA was used due to the violation of variance homogeneity assumption.

**Table 3:** Factors associated with all-cause mortality in a Cox regression model.

Variables	Hazard Ratios	95% Confidence Interval	*p*-Value
COPD groups			
NPV + ND	1		
NPV + D	2.02	0.89–4.60	0.09
Non-NPV + ND	2.42	1.13–5.19	0.02
Non-NPV + D	2.52	1.11–5.72	0.03
Age (year)	1.02	0.99–1.05	0.19
Gender			
Female	1		
Male	3.57	0.81–15.7	0.09
Smoking			
No	1		
Yes	1.37	0.76–2.48	0.30
FEV_1_ (%) per 10% decrease	1.01	0.99–1.02	0.06
Annual admission rate	1.13	0.85–1.50	0.41
Charlson index			
≤2	1		
≥3	1.17	0.61–2.26	0.63
6MWD per 10-m increase	0.98	0.96–1.00	0.03
Body mass index	0.99	0.93–1.05	0.73
Nadir saturation during 6MWT			
SpO_2_: ≥90	1		
SpO_2_: 80–89	2.67	1.35–5.28	<0.01
SpO_2_: <80	3.13	1.33–7.36	<0.01

NPV: negative pressure ventilation, D: desaturation, ND: non- desaturation, CI: confidence interval, FEV_1_: forced expiratory volume in the first second, 6MWD: 6-min walk distance, 6MWT: 6-min walk test, SpO_2_: oxygen saturation by pulse oximetry.

## Supplementary Materials

The following are available online at https://www.mdpi.com/2077-0383/8/4/562/s1, Figure S1: EtCO_2_ and heart rate significantly decreased, and SpO_2_ remained over 90% during the NPV titration process, Figure S2: Transition dyspnea index score was evaluated by the day after patients underwent negative pressure ventilation (NPV) for 60 min, Table S1: Mortality risks during 8 years in the four groups, adjusted by confounders, Table S2: Regression coefficients for the mixed-model repeated-measure models for FEV_1_ and FEV_1_ %, Table S3: Regression coefficients for the mixed-model repeated-measure models for 6-min walking distance, Table S4: Regression coefficients for the mixed-model repeated-measure models for Distance-saturation product (M%).
