# Supplementary material for: Maintenance Negative Pressure Ventilation Improves Survival in COPD Patients with Exercise Desaturation
Source: J Clin Med. 2019 Apr 25;8(4):562. doi: 10.3390/jcm8040562 (PMC6518192; doi:10.3390/jcm8040562)
Supplement: Supplementary file 1 [file jcm-08-00562-s001.pdf]

Journal of
Clinical Medicine

MDPI

Article

Maintenance Negative Pressure Ventilation Improves Survival in COPD Patients with Exercise Desaturation

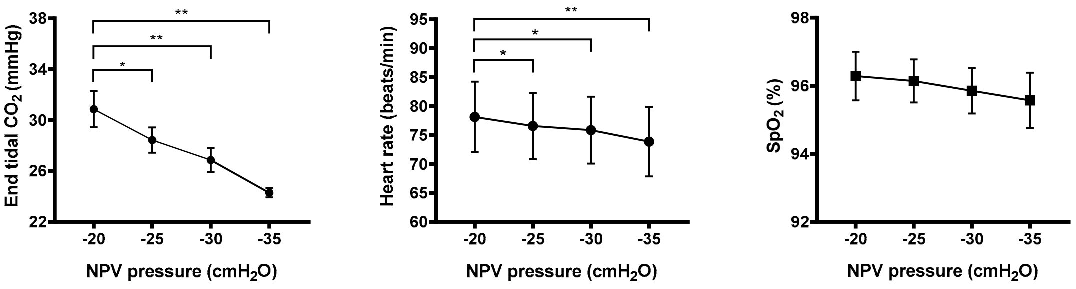

Figure S1. EtCO2 and heart rate significantly decreased, and SpO2 maintained over 90% during the NPV titration process. *$p < 0.05$, ** $p < 0.01$ was compared to the pressure level of -20 cmH_2O.

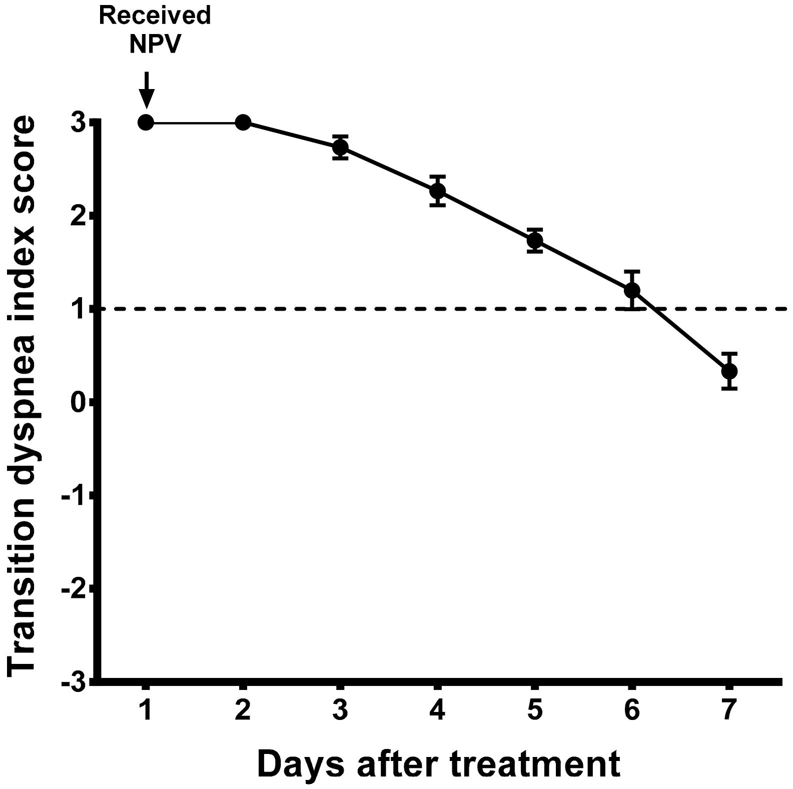

Figure S2. Transition dyspnea index score was evaluated by the day after patients underwent negative pressure ventilation (NPV) for 60 minutes. The response of NPV confirmed a 1-unit change in the TDI score as being clinically important.

Table S1. Mortality risks during 8 years in the four groups adjusted by confounders.

Variables	3 year HR(95%CI)	P value	5 year HR(95%CI)	*p* value	8 year HR(95%CI)	*p* value
NPV-ND	1		1		1	
NPV-D	9 (1-70)	0.0431	5 (1-12)	0.0014	3 (2-7)	0.0022
Non-NPV-D	11 (1-88)	0.0255	5 (2-14)	0.0004	4 (2-9)	0.0002
Non-NPV -ND	6 (0.7-49)	0.0943	2 (0.8-6)	0.1149	3 (1-5)	0.0171

Adjusted risk compared to NPV-ND group. HR: hazard ratio. NPV: negative pressure ventilation, D: desaturation, ND: non-desaturation.

Table S2. Regression coefficients for the mixed-model repeated-measure models for FEV_1 and FEV1 %.

Solution for Fixed Effects: forced expiratory volume in one second (FEV_1)						
**Effect**	**Group**	**Estimate**	**Standard error**	**DF**	**t value**	*p* value
Intercept		1356.200	6405.530	337	0.21	0.833
time		-0.360	0.648	336	-0.56	0.579
group	Non-NPV + D	-404.740	8736.490	1804	-0.05	0.963
group	Non-NPV + ND	-157.610	8924.580	1804	-0.02	0.986
group	NPV + D	-404.900	8976.560	1804	-0.05	0.964
group	NPV + ND	0	.	.	.	.
time*group	Non-NPV + D	-1.890	0.741	1804	-2.55	0.0109
time*group	Non-NPV + ND	-1.396	0.727	1804	-1.92	0.0549
time*group	NPV + D	-0.137	0.738	1804	-0.19	0.8529
time*group	NPV + ND	0	.	.	.	.
time*time		-0.013	0.007	1804	-1.86	0.0636
Solution for Fixed Effects: forced expiratory volume in one second (FEV1 %)						
Effect	Group	Estimate	Standard error	DF	t value	*p* value
Intercept		62.082	2.279	337	27.24	<.0001
time		0.132	0.038	336	3.49	0.0006
group	Non-NPV + D	-18.190	3.113	1804	-5.84	<.0001
group	Non-NPV + ND	-6.624	3.165	1804	-2.09	0.0365
group	NPV + D	-18.688	3.191	1804	-5.86	<.0001
group	NPV + ND	0	.	.	.	.
time*group	Non-NPV + D	-0.107	0.041	1804	-2.61	0.0091
time*group	Non-NPV + ND	-0.124	0.039	1804	-3.16	0.0016
time*group	NPV + D	-0.030	0.040	1804	-0.74	0.4566
time*group	NPV + ND	0	.	.	.	.
time*time		-0.002	0.000	1804	-5.52	<.0001

NPV: negative pressure ventilation, D: desaturation, ND: non-desaturation.

Table S3. Regression coefficients for the mixed-model repeated-measure models for 6 minute walking distance.

Solution for fixed effects: 6 minute walking distance (meter, M)						
**Effect**	**Group**	**Estimate**	**Standard error**	**DF**	**t value**	*p* value
Intercept		389.300	10.377	337	37.51	<.0001
time		0.944	0.283	316	3.33	0.0010
group	Non-NPV + D	-18.213	14.423	1527	-1.26	0.2069
group	Non-NPV + ND	-26.656	14.464	1527	-1.84	0.0655
group	NPV + D	-29.656	14.593	1527	-2.03	0.0423
group	NPV + ND	0	.	.	.	.
time*group	Non-NPV + D	-1.744	0.335	1527	-5.20	<.0001
time*group	Non-NPV + ND	-0.920	0.330	1527	-2.79	0.0054
time*group	NPV + D	-1.084	0.324	1527	-3.34	0.0009
time*group	NPV + ND	0	.	.	.	.
time*time		-0.019	0.003	1527	-6.04	<.0001

NPV: negative pressure ventilation, D: desaturation, ND: non-desaturation.

Table 4. Regression coefficients for the mixed-model repeated-measure models for Distance-saturation product (M%).

**Solution for Fixed Effects: Distance-saturation product (M%)**						
**Effect**	**Group**	**Estimate**	**Standard error**	**DF**	**t value**	*p* value
Intercept		354.040	9.342	336	37.90	<.0001
time		0.411	0.249	316	1.65	0.0998
group	Non-NPV + D	-48.799	13.014	1527	-3.75	0.0002
group	Non-NPV + ND	-25.514	13.019	1527	-1.96	0.0502
group	NPV + D	-62.926	13.135	1527	-4.79	<.0001
group	NPV + ND	0	.	.	.	.
time*group	Non-NPV + D	-1.222	0.291	1527	-4.20	<.0001
time*group	Non-NPV + ND	-0.773	0.284	1527	-2.72	0.0066
time*group	NPV + D	-0.606	0.281	1527	-2.16	0.0308
time*group	NPV + ND	0	.	.	.	.
time*time		-0.01391	0.002758	1527	-5.04	<.0001

NPV: negative pressure ventilation, D: desaturation, ND: non-desaturation.
